# An advanced in vitro bladder cancer model integrating bladder cancer spheroids into a healthy human urothelium for preclinical therapeutic testing

**DOI:** 10.1038/s41416-026-03476-0

**Published:** 2026-06-02

**Authors:** Benjamin O. Murray, Jinhui Gao, Laia Pasquina-Lemonche, Katherine Swarbrick, Guy Simpson, Mark A. Chambers, Hardev Pandha, Alex Freeman, Jennifer L. Rohn

**Affiliations:** 1 Centre for Kidney and Bladder Health, Division of Medicine, University College London, London, UK; 2 School of Biosciences, University of Sheffield, Sheffield, UK; 3 Histopathology Department, Camelia Botnar Laboratories, Great Ormond Street Hospital for Children NHS Foundation Trust, London, UK; 4 Department of Microbes, Infection and Immunity, School of Biosciences, University of Surrey, Guildford, UK; 5 Department of Oncology, Faculty of Health and Medical Sciences, Leggett Building, University of Surrey, Guildford, Surrey UK; 6 Department of Pathology, University College London Hospitals NHS Foundation Trust, London, UK

**Keywords:** Bladder cancer, Cancer models, Targeted therapies

## Abstract

**Background:**

Despite therapy advances, better solutions for refractory bladder cancer remain an unmet need. Human cell-based models may aid in better treatment translation. We introduce 3D-UHU-TU, a bladder cancer microtissue model which incorporates spheroids derived from low- and high-grade human bladder cancer cell lines (RT112 and T24 respectively) into a healthy human urothelium.

**Methods:**

After model characterisation with histopathology and immunofluorescence microscopy, we trialled both the conventional chemotherapeutic Mitomycin C (MMC), and a novel herpes simplex oncolytic virus (oHSV-GFP), each assessed using confocal microscopy and cytotoxicity assays.

**Results:**

We observed correct expression of E- and N-Cadherin, CK7, CK20, GATA3 and Ki-67, alongside invasion and migration phenotypes. MMC treatment caused cell lysis and nuclear damage in cancer spheroids in both low- and high-grade models, with minimal damage to the surrounding healthy urothelium, and a significant increase in cleaved caspase 3 in low-grade models. oHSV-GFP co-localised in cancer spheroids and induced syncytia, spheroid disaggregation and cytotoxicity with minimal to no co-localisation or cytotoxicity in the healthy urothelium.

**Conclusion:**

3D-UHU-TU model is useful for testing both treatment safety and efficacy on different grades of bladder cancer. Future use of primary tumour spheroids in place of cell lines may allow a personalised medicine approach.

**Figure:**
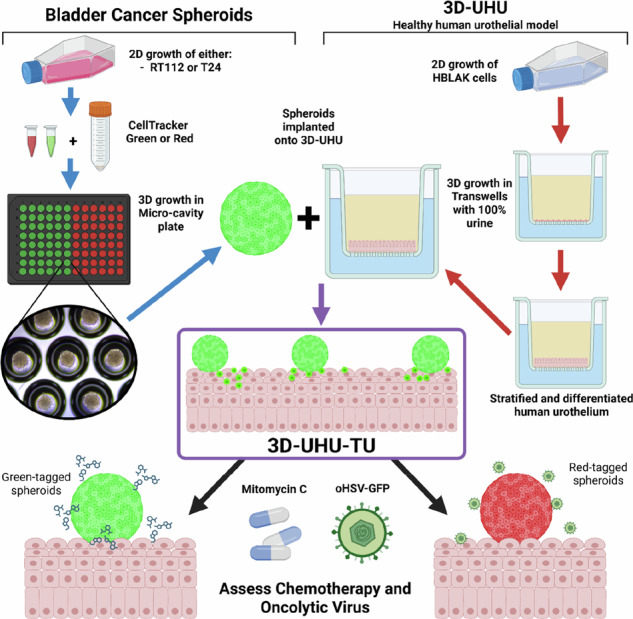

## Introduction

Bladder cancer is one of the most expensive cancers to treat [1, 2], and cases and deaths continue to rise [3, 4]. Due to its molecularly heterogenous nature with various transcriptional, mutational and lineage signatures [5], tumour type-specific treatments are limited, and outcomes have not improved for the last four decades [6], lagging behind progress with many other cancers [7]. More recent advances in immunotherapies [8] and antibody-drug conjugates, for example enfortumab vedotin [9], are promising, but not all patients respond to them, and their adverse effects can be limiting.

Urothelial carcinomas are described clinically by two stage groupings: non-muscle invasive (NMIBC) and muscle-invasive bladder cancer (MIBC). NMIBC (clinical stage Tis, Ta and T1) comprises ~75% of cases, further separated into low-, intermediate-, high- and very high risk categories, for which a limited number of treatments can be implemented [10, 11]. The chance of recurrence of high-risk NMIBC five years after surgical resection is ~69.5%, with up to ~22.8% progressing to MIBC [12]. First-line treatment for MIBC includes neoadjuvant chemotherapy followed by radical cystectomy or chemoradiotherapy. The patient impact can be significant, with overall survival post-treatment at 49% after 5 years and 36% at 10 years [13], with considerable detriment to quality-of-life [14, 15]. This highlights the importance of developing new therapies and models to evaluate them, especially at the NMIBC stage before progression has occurred [16].

The most widely used preclinical models for bladder cancer are orthotopic and xenograft murine models. However, working with animals is labour-intensive, ethically challenging, expensive and subject to considerable regulation. Other disadvantages include the length of time needed to establish disease (4–6 months) and, in the case of PDX models, poor implantation rates [17]. More profoundly, there are concerns about physiological inaccuracies caused by species difference in the surrounding urothelial environment [18]. As the method of tumorigenesis and the tumour microenvironment greatly influence the molecular subtype, in turn influencing cancer development and treatment response [19], human cancer cells implanted into a mouse may not respond as they would in a human context [20]. This highlights the need for developing complementary human-based approaches.

Recently, our laboratory developed a 3D urine-tolerant human urothelium model (3D-UHU) used for research into urinary tract infections [21, 22]. In this microtissue model, cells stratify to 5–7 cell layers (as opposed to the mouse urothelium, which only consists of three) with terminally differentiated umbrella cells. Urothelial biomarkers are also correctly expressed (claudins, uroplakins, zonula occludens-1 [ZO-1], cytokeratins, Toll-like receptors, E-Cadherin) with the formation of a luminal glycosaminoglycan layer and strong barrier function. Importantly, it is fully urine-tolerant for weeks, allowing for experimentation in the native environment, which may affect both tumour and normal urothelial gene expression as well as response to chemotherapy.

Here, we created and characterised hybrid tumour/healthy cell models using the 3D-UHU model co-cultured with bladder cancer spheroids derived from urothelial carcinoma cell lines RT112 (low grade) and T24 (high grade), and tested their utility. After characterising the 3D-UHU-Tumour (3D-UHU-TU) models for markers involved in diagnosis, adhesion, invasion/migration and proliferation, we assessed them as a testbed for treatment with the common post-operative intravesical chemotherapeutic, mitomycin C (MMC), as well as a novel oncolytic virus. Our results show that the models hold promise both for understanding the interactions between cancer and healthy cells and for trialling potential therapies in a manner far simpler, quicker and cheaper than animal experimentation, thereby adhering to the 3Rs principles.

## Methods and materials

### Cell culture

Human HBLAK cells (CELLnTEC, Switzerland) were cultured as described previously [21]. Briefly, HBLAK cells were maintained in T150 flasks in 2D Prime media (CnT-PR; CELLnTEC, Switzerland) at a seeding density of 3 × 10^5^. Cells were incubated at 37 °C at 5% CO_2_ for 5–6 days with media changed every 2–3 days until 80–90% confluent. Cells were detached using Accutase solution (CELLnTEC, Switzerland) and collected by centrifugation at 300xg for 5 min. The cells were either passaged up to P13 or used for 3D cell culture (from P8-P13).

The RT112 (low-grade) human bladder cancer cell line, previously isolated from a female with transitional cell carcinoma, was kindly provided by Nenna Kanu and Mark Linch (University College London, UK). The T24 (high-grade) bladder cancer cell line, previously isolated from an 81-year-old female, was purchased from ATCC. The stage of these cell lines is hard to decipher but origins have been reported for RT112 [23] and T24 [24], and both have been used in the past for the creation of orthotopic mouse models [25]. RT112 and T24 were cultured in complete DMEM (ThermoFisher Scientific, UK) with 10% foetal bovine serum (FBS; ThermoFisher Scientific, UK) supplemented with Pen/Strep solution (100U/ml; Merck, UK; A5955). Both lines were incubated at 37 °C with 5% CO_2_. Cells were harvested for passage or spheroid generation at ~90% confluency and detached with 0.25% Trypsin-EDTA (ThermoFisher Scientific, UK) and collected by centrifugation at 300 × *g* for 5 min. Cell lines are STR profiled, were tested for mycoplasma and all were mycoplasma free. No patient material was used throughout the study.

### 3D-UHU model production

3D-UHU microtissue models were created as described previously [21, 22]. Briefly, HBLAK cells were added to 0.4 µm polycarbonate Transwell membrane inserts (Corning, USA) at a seeding density of 4.5 × 10^5^ with 400 µl 2D Prime media in apical chambers and 1.5 ml 2D Prime media in basal chambers. After 100% confluency (48 h), pre-warmed 3D Prime media (CnT-PR-3D; CELLnTEC, Switzerland) was added to apical and basal chambers. After at least 72 h, Transwells were transferred to 12-well Thincert® plates (Greiner Bio-One, UK), and 300 µl of filtered pooled-gender human urine (BioIVT, UK) from healthy donors were added to the apical chambers and 4.5 ml 3D Prime media to basal chambers. Media and urine were changed every 3–4 days for 14–18 days. All human urine used in this study was commercially available from BioIVT pooled together from a minimum of 5 male and 5 female donors.

### RT112/T24 spheroid generation and growth curves

Trypsinised RT112 and T24 cells were collected and resuspended in complete culture media separately. CellTracker Green CMFDA dye (Invitrogen, UK) was added to centrifuged cells according to the manufacturer’s instructions, which were then seeded into Elplasia microspheroid plates (Corning, UK) at 1000 cells/microcavity in 200 µl media. The manufacturer’s guidelines state there are approximately 79 microcavities per 96-well. Plates were incubated at 37 °C with 5% CO_2_ for 2–4 days. Spheroids were imaged using a Leica DMi1 inverted microscope and captured using LAS X software V5.0.2.24429. Images were analysed using Image J V1.54 f [26].

### 3D-UHU-TU establishment: Incorporation of cancer spheroid onto 3D-UHU models

On the day of spheroid implantation, a full media/urine change was performed on mature 3D-UHU models. For every condition, we deposited an entire well of spheroids onto one separate 3D-UHU model. Spheroids were harvested using a wide-bore 200 µl tip. 150 µl was pipetted up and down gently to dislodge spheroids and then collected. To ensure all spheroids were collected, the microcavities were checked under the microscope to confirm that they were empty. We also conducted a macroscopic inspection of the spheroids in the pipette tip to ensure spheroids were present. The suspension was deposited into the apical urine slowly and at different positions. The models were then incubated overnight at 37 °C. The next day, urine/DMEM solution was changed to fresh 100% urine.

### Calculation of implantation rate of cancer spheroids onto 3D-UHU

To quantify the implantation rate of cancer spheroids onto the 3D-UHU model, RT112 or T24 spheroids were pre-labelled with CellTracker™ Green CMFDA dye and 3D-UHU-TU models were established as described above. Following spheroid seeding, co-cultures were incubated overnight under standard culture conditions to allow spheroid attachment and integration, after which models were fixed in 4% methanol-free formaldehyde (FA; Fisher Scientific, UK) prepared in phosphate-buffered saline (PBS; ThermoFisher Scientific, UK).

Confocal imaging was performed using a Leica SP8 laser-scanning confocal microscope. To enable comprehensive surface coverage of the 3D-UHU, a spiral scanning acquisition mode was employed, generating a continuous top-down overview of the surface of 3D-UHU-TU. CellTracker Green-positive cancer spheroids visible on the surface were manually counted. The implantation rate was calculated as the number of implanted spheroids detected on the 3D-UHU surface divided by the total number of spheroids initially seeded (approximately 79 spheroids) as a percentage.

### Immunofluorescence staining

Immunofluorescence staining was performed as previously described [22], with some minor changes. Briefly, samples were fixed overnight at 4 °C in 4% FA. Transwell membranes were excised using a scalpel and transferred to a 24-well plate. Cells were permeabilised with 0.2% Triton X-100 (Sigma Aldrich, UK) for 45 min, followed by a PBS wash. Blocking was carried out at room temperature (RT) for 1 h using PBS containing 5% normal goat serum (NGS; ThermoFisher Scientific, UK) and 1% bovine serum albumin (BSA; Merck, UK). After removing the blocking buffer, samples were washed once with PBS containing 1% BSA. Primary antibodies (Table S1) were diluted in PBS with 1% NGS and 1% BSA, and incubated overnight at 4 °C. Samples were then washed three times with PBS containing 1% BSA. Secondary antibodies (Table S1) were applied for 1 h at RT, followed by three PBS washes.

For nuclear and cytoskeletal staining, samples were incubated with 4’,6-diamidino-2-phenylindole (DAPI) and Phalloidin-555 or -647 (ThermoFisher Scientific, UK; Table S1) for 1 h at RT. After staining, samples were mounted onto glass slides using ProLong Diamond antifade mounting medium (Invitrogen, UK), sealed with nail varnish, and left to dry overnight at RT. Images were acquired using a Leica SP8 confocal microscope and analysed as described above.

### Immunohistochemistry

3D-UHU-TU models were fixed in 10% neutral buffered formalin for no longer than 24 h, inked with 0.1% safranin O, bisected, processed using the Leica Peloris II Tissue Processor (Leica Biosystems) and embedded on edge into paraffin blocks using the Tissue-Tek TEC 6 embedding centre (Sakura Finetek, USA) with the cut surface of bisected wells facing down. 3 µm sections were obtained with Leica Histocore Autocut microtome (RM225, Leica Biosystems), mounted on TOMO adhesive slides (SolMedia) and baked at 60 °C before staining with haematoxylin and eosin (H&E) on the Leica Autostainer X (Leica Biosystems). Immunohistochemistry (IHC) staining was performed on the Leica Bond Max (Leica Biosystems) and Ventana Benchmark Ultra (Roche) automated immunostaining platforms.

### Mitomycin C treatment

Mitomycin C (MMC) (11435-25mg-CAY; Cambridge Bioscience, UK) stocks were diluted in pre-warmed 0.85% saline (Fisher Scientific, UK) to 0, 10, 25 and 50 µg/ml working solutions, then added to either RT112/T24 spheroids, 3D-UHU or to complete 3D-UHU-TU models. These were incubated for 2 h at 37 °C. MMC solution was then aspirated out and fresh cell culture media or urine was added to the relevant models.

For spheroid growth curves, MMC treated (10 µg/ml only) and untreated spheroid diameters were measured as described above over 3 days post-treatment. Next, an LDH assay (Invitrogen, UK) was used to determine cytotoxicity of each separate component of the 3D-UHU-TU models. Supernatants were collected 24 h post-treatment and used for subsequent LDH assays. Complete 3D-UHU-TU models were treated and incubated for 24 h before being fixed, stained and imaged with the SP8 confocal microscope as described above. These experiments were repeated at least two additional times independently.

### Image analysis MMC treatment experiments

Image analysis of MMC treatment experiments was performed via a custom-made ImageJ macro named “FluorMask3D” (available from 10.5281/zenodo.19582036). The macro was comprised of a combination of existing FIJI language functions and commands to perform a background correction using maths>subtract (mean value of a slice with only background); followed by filtering of the image with a Gaussian blur (2 sigma) and reducing of the pixels. Next, the 3D image was binarized by creating a stack of masks (where the threshold was maintained constant for all the slices by using the slice that was approximately in the middle of the spheroid as a reference). Finally, a custom-made function named “Fit Double Logistic” was created to find the steeper points in the “double logistic”-shape-like curve from the total area vs. slice graph plotted by FIJI from the green channel. The green channel indicated where the cells from the cancer spheroid were located; finding the region of interest that included only the spheroid was crucial to be able to detect the fluorescence intensities corresponding only to the spheroid region from the other channels (yellow and cyan). The resultant ratio between yellow and cyan was the relative fluorescence of cleaved caspase-3 in each sample and plotted. See Supplementary Fig. 5 (S5) for more details. Graphs were generated using GraphPad Prism V9.1.2 – V10.4.2. Two-way ANOVA statistical analysis was performed with Šídák’s multiple comparison test.

### oHSV-GFP treatment and co-localisation in 3D-UHU and 3D-UHU-TU models

Oncolytic Herpes Simplex Viruses (oHSV) were described previously [27]. Multiplicity of infection (MOI) was calculated combining the total umbrella cell layer (~30,000 cells [21]) and the average number of spheroids implanted (~50 spheroids at 1000 cells per spheroid) with proliferation of cancer spheroids taken into account (~60,000 total cancer cells). Models were treated at an MOI of 0.05 (4.5 × 10^3^ pfu/well), incubated for 30 min at 37 °C and the viral supernatant was discarded. Next, the models were washed once gently with pre-warmed PBS and replaced with fresh urine. 3D-UHU-TU were fixed in 4% FA overnight at 4 °C after 24, 48, 72, 96 and 120 h post-treatment. Urine was replaced every 24 h in wells that were not fixed to simulate urination. Models were permeabilised, stained with DAPI and Phalloidin-647, and imaged with Leica SP8 confocal microscope and analysed as above.

### oHSV-GFP cytotoxicity on cancer spheroids and 3D-UHU-TU models

Both cancer spheroids alone, 3D-UHU and 3D-UHU-TU models were established and either treated with oHSV-GFP at MOI 0.05 or untreated as described above. The LDH assay was performed on media or urine at 24, 48, 72, 96, 120 h post viral treatment as above. In parallel, models were fixed at each time point at 4 °C overnight as above. Urine was changed every day to simulate urination as before in models that were not fixed. Media was also replaced in the cancer spheroids. Fixed 3D-UHU-TU models were permeabilised, stained with DAPI and Phalloidin-647, imaged with confocal microscopy and analysed as described above. Statistical analysis was performed with the two-way ANOVA test with Tukey’s multiple comparisons test. All experiments reported use at least 3 biological and technical replicates.

## Results

### Establishment of cancer spheroids and 3D-UHU-TU model

**Fig. 1:**
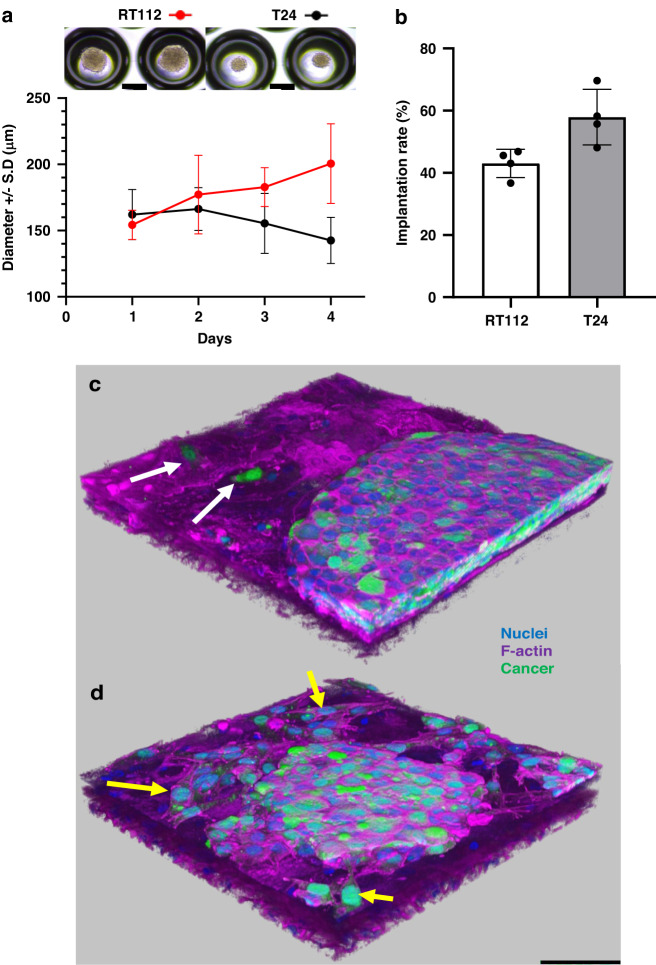
3D-UHU-TU Generation. (**a**; Top) Day 4 RT112 and T24 spheroids grown in DMEM. Scale bars = 200 µm. (**a**; bottom) Growth curves of isolated spheroids, generated from 12 technical replicates from 3 independent experiments. **b** Implantation rate of spheroids onto 3D-UHU model. Optimised 3D-UHU-TU with (**c**) RT112 and (**d**) T24 spheroids tagged with CellTracker CMFDA probes (green). RT112 spheroids flattened and maintained their spherical shape with some migrating cells on top of urothelial surface, which integrated into the urothelium (white arrows). T24 spheroids were smaller and exhibited more migration of cells at spheroid edge (yellow arrows). Representative images from 3 independent experiments. Scale bar = 100 µm.

To create a model combining the human tumour cells into the healthy 3D-UHU microenvironment, we chose human bladder cancer cells lines RT112 (low grade), and T24 (high grade) for tumour spheroid generation. These cell lines have been frequently used in orthotopic mouse models [25], making our data comparable with the current literature. The resulting spheroids reached an average diameter of 200 µm and 140 µm respectively (Fig. 1a).

3D-UHU models contained 5–7 layers of urothelial cells, and all had a differentiated umbrella cell layer (data not shown). When incorporated onto the 3D-UHU surface, RT112 and T24 spheroids had a successful implantation rate of 43% and 57% respectively (Fig. 1b) and were maintained in 100% urine for at least 24 h. Both RT112 and T24 spheroids exhibited different phenotypes. RT112 spheroids were larger and maintained their spherical shape when integrated (Fig. 1c). In contrast, T24 spheroids readily spread out over the surface of the urothelium with the spheroid edges migrating away from the main spheroid mass and integrating extensively into the top layer of the urothelium (Fig. 1d). The flattened nature of the spheroids was caused by the pressure of coverslips needed for microscopy. Larger RT112 spheroids (>400 µm) were also successfully incorporated (Fig. S1).

### Characteristics of 3D-UHU-TU models via immunohistochemistry and immunofluorescence

To further characterise the 3D-UHU-TU models, we used microscopy to inspect the microtissue for a variety of key proteins after co-culture of the tumour spheroids with the 3D-UHU urothelium ranging from 1 to 6 days in 100% urine.

#### E- and N-Cadherin expression

**Fig. 2:**
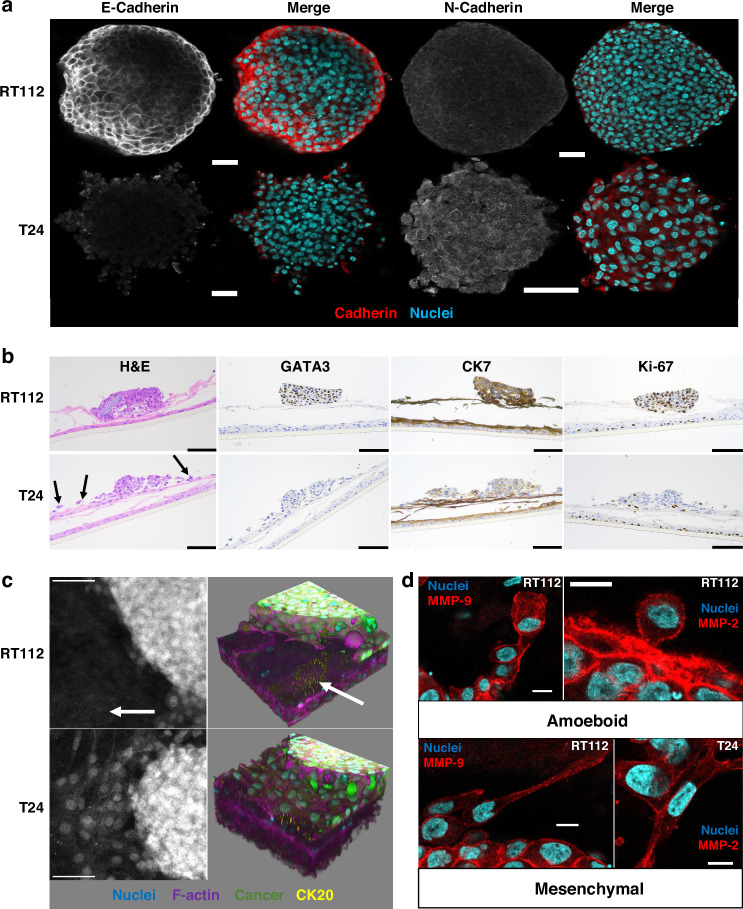
Expression of various markers in 3D-UHU-TU. **a** E- and N-Cadherin (grey) in 3D-UHU-TU in 100% urine environment 2 days post-implantation (3D-UHU layer below plane of focus). For merged images, Cadherin = Red, nuclei = cyan. Scale bars = 50 µm. **b** H&E and IHC in models maintained for 6 days in 100% urine environment. Images are representative of 3 separate biological replicates. Bars = 50 µm. **c** CK20 expression (left panels; greyscale) with 3D merges. Models were stained for F-Actin (magenta), which highlights the actin cytoskeleton, useful for providing an overview of cell morphology, nuclei (cyan), cancer spheroid (green) and CK20 (yellow). CK20 on urothelium are highlighted with white arrows. Models maintained for 6 days in 100% urine environment. **d** Migration phenotypes magnified from Fig. S2B, C. MMP-2 & -9 (red) and nuclei (cyan) stained. Scale bars = 10 µm. Representative images from 3 independent experiments.

Cadherins are cell-to-cell adhesion mediators expressed in bladder cancer and known indicators of cancer progression [28]. E-Cadherin is expressed in healthy epithelium and Ta tumours, but switch to N-Cadherin in more invasive, high-grade tumours [29]. We examined 3D-UHU-TU models for expression of E- and N-Cadherin (Fig. 2a). As expected, E-Cadherin was expressed 2 days post-implantation in the low-grade RT112 spheroids (Fig. 2a), localised correctly at cell-cell junctions, and in the healthy urothelial component, particularly in the superficial umbrella cell layer (Fig. S2A) and similarly enriched at junctions. In the T24 spheroids, as expected for a high-grade cancer, E-Cadherin was largely lacking, with possible low expression on spheroid edges. Reciprocally, N-Cadherin was not expressed in the RT112 spheroids, but was in T24 spheroids (Fig. 2A).

#### Diagnostic Biomarkers GATA3, CK7 and CK20

To determine whether the 3D-UHU-TU models retained spatial markers reminiscent of patient-derived bladder cancer tissue, we first employed immunohistochemistry of several key proteins, alongside haematoxylin and eosin (H&E) staining, using fixed, paraffin-embedded sections, orthogonally bisecting the area of the microtissue containing a spheroid in 3D-UHU-TU models maintained for 6 days post-implantation in a 100% urine environment.

The H&E preparations revealed the compact structure of RT112 spheroids (Fig. 2b), similar to the confocal micrographs (Fig. 1c and Fig. S1). In contrast, the T24 spheroids exhibited a more spreading pattern, with cell migration from the spheroid mass (black arrows; Fig. 2b) consistent with the metastatic features of this high-grade cell line (as seen similarly in Fig. 1d).

Next, we inspected markers commonly used in bladder cancer diagnosis. GATA3 is a transcription factor typically found in the nuclei of lower-grade bladder cancer but down-regulated in higher-grade cancers, making it an important diagnostic marker for tumour grading [30]. In the 3D-UHU-TU models, GATA3 was expressed in the low-grade RT112 cancer spheroid but not in the high-grade T24 cancer spheroid (Fig. 2b), nor in their healthy urothelial components.

Expression of CK7 is commonly used in the diagnostic laboratory and is expected to be expressed in all layers of the urothelium and in cancer. Indeed, CK7 was strongly expressed in both model compartments (Fig. 2b). CK20 can be expressed in both low- and high-grade bladder cancer tissue [31], and expressed on the apical surface of the healthy urothelium [32] and on the 3D-UHU model [21]. In both RT112 and T24 models, CK20 was detected in the tumour (Fig. 2c) and surrounding urothelium (orange arrow; Fig. 2c).

#### Proliferation marker – Ki-67

Ki-67 is a widely used proliferation marker in cancer cell biology, being a key cell cycle protein upregulated in proliferating cells and downregulated in resting cells [33]. In both models, Ki-67 expression was seen in the basal cell proliferative compartment of the surrounding normal urothelium, as well as in some intermediate cells, as expected in the transitional urothelium. In the low-grade RT112 model (Fig. 2b), robust Ki-67 staining was seen in the tumour mass, consistent with the observed growth and migration of the cancer spheroid in the urine environment after 6 days post-integration. In contrast, the T24 model exhibited limited Ki-67 expression within the cancer spheroids (Fig. 2b and S3; right of the orange dotted line). However, T24 cells migrating away from the spheroid mass expressed Ki-67 2 days post-integration (Fig. S3).

#### Invasion and migration markers – MMP2/9

We next investigated invasion and migration phenotypes. In RT112 spheroids, some peripheral cells exhibited a mix of amoeboid and mesenchymal migration phenotypes (Fig. 2d). Amoeboid migration phenotypes normally consist of a rounded morphology with dynamic extensions [34], while mesenchymal phenotypes are more elongated with prominent nuclei [35]. In T24 spheroids, peripheral cells only expressed mesenchymal migration phenotypes (Fig. 2d), as expected for a higher-grade cancer. We also observed a collective migration phenotype [36] in the RT112 spheroid (magenta arrows; Fig. S2C). Matrix Metalloproteinase (MMP) -2 and -9 (also known as gelatinase A and B respectively) have been shown to be important for cancer progression, particularly for immunomodulation, cell migration, invasion and morphogenesis of different cancers [37], and were both expressed on both RT112 and T24 cancer spheroids 24 h post-integration (Fig. S2B and C).

### 3D-UHU-TU as a testbed for treatments

#### Mitomycin C treatment has tumour-specific efficacy in 3D-UHU-TU models

To validate 3D-UHU-TU as a potential platform for treatment testing, we assessed Mitomycin C (MMC), one of the most commonly used chemotherapeutic agent for treatment of NMIBC [38], which alkylates DNA, causing double-strand breaks that initiate apoptosis [39].

**Fig. 3:**
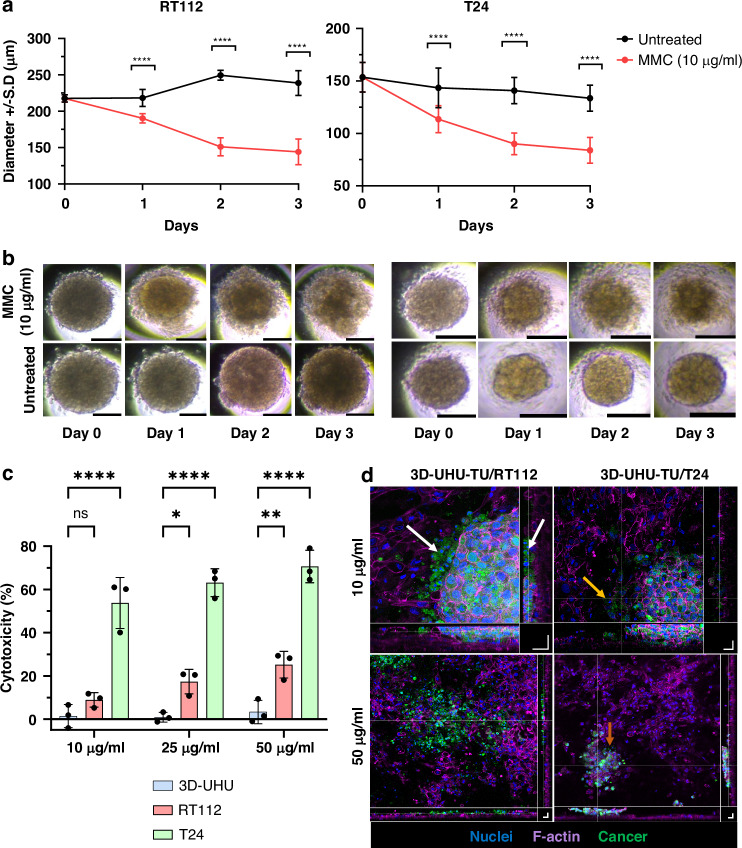
MMC treatment of 3D-UHU-TU components and complete models. **a** RT112 and T24 spheroid growth curves over 3 days treated with 10 µg/ml MMC for 2 h. Results are from 12 technical replicates from 3 biological replicates. ****P = < 0.0001. **b** RT112 and T24 spheroid morphology after 2 h treatment with 10 µg/ml MMC (top row) over 3 days. Untreated spheroids were also measured over 3 days (bottom row). Scale bars = 100 µm. **c** LDH Cytotoxicity of separate components of the 3D-UHU-TU model treated with different doses of MMC for 2 h. Results are from 3 biological replicates. *P = < 0.05; **P = < 0.01; ****P = < 0.0001. **d** MMC treatment of 3D-UHU-TU models. Each large image is the top-down view, while the right and bottom zones past the thick white line indicate the cross-sectional view at the site marked with the fine white crosshairs in the main image. Models were treated for 2 h and recovered in urine for 24 h. The RT112 spheroid component lost spheroid structure and exhibited cellular lysis and damage (top left panel, white arrows) at 10 µg/ml with urothelial layer still intact. At 50 µg/ml, the RT112 model cancer spheroid had disintegrated (bottom left panel). 3D-UHU component was still intact. In the T24 models, spheroids also showed cellular damage of migrating cells (top right panel, orange arrow) at 10 µg/ml MMC. At 50 µg/ml, the T24 spheroid has decreased in size with visible damage observed (bottom right panel, brown arrow). Scale bars = 20 µm. Nuclei=blue; F-actin=magenta; cancer cells=green. Images representative of 3 independent experiments.

First, we assessed MMC treatment of 10 µg/ml for 2 h on cancer spheroids only and found a significant decrease in cancer spheroid diameter over 3 days compared with untreated spheroids (Fig. 3a). This was also confirmed morphologically, with disaggregation observed in both spheroid types treated with MMC (Fig. 3b).

Next, we wanted to see if larger doses of MMC would show differences on the separate 3D-UHU-TU model components. Each component was treated with 0, 10, 25 and 50 µg/ml MMC for 2 h and allowed to recover for 24 h after treatment. The LDH assay was used to establish cell cytotoxicity. For the 3D-UHU component, treatment up to 50 µg/ml produced 3.29% cytotoxicity, indicating that the 3D-UHU component was mostly resistant to MMC treatment at the highest tested dose (Fig. 3c). RT112 spheroids, on the other hand, showed a significant increase in cytotoxicity compared with that seen in 3D-UHU at 25 and 50 µg/ml, whereas T24 had a significant increase of cytotoxicity with all doses compared with 3D-UHU (Fig. 3c).

After treatment, we evaluated cytotoxicity via immunofluorescence staining on the complete 3D-UHU-TU models. We used a low- (10μg/ml) and high-dose (50 µg/ml) treatment for two hours; afterwards, the treatment was replaced with urine and the models were allowed to recover for 24 h before assessment. For the RT112 models (Fig. 3d; left panels), MMC caused damage to the spheroids in a dose-dependent manner. While the saline control did not induce morphological damage nor affect the spheroid structure (Fig. S4; left panel), at 10 µg/ml MMC, the spheroid structure was still intact, but extensive cellular lysis and nuclear damage were evident (Fig. 3d, top left panel, white arrows). At 50 µg/ml, the RT112 spheroids appeared to be completely disaggregated, including areas buried within the urothelium (compared from orthologous views; Fig. 3d). The healthy urothelium, on the other hand, was still intact (Fig. 3d, non-green cells).

In the T24 models, saline treatment again induced no morphological damage to the model (Fig. S4; right panel). At 10 µg/ml MMC, the cancer spheroid maintained its structure with some cellular damage to migrating cells (Fig. 3d; top right panel, orange arrow), comparable to those observed with RT112. At 50 µg/ml, the cancer spheroids were much smaller, and some cellular damage was observed (Fig. 3d, bottom right panel, brown arrow).

**Fig. 4:**
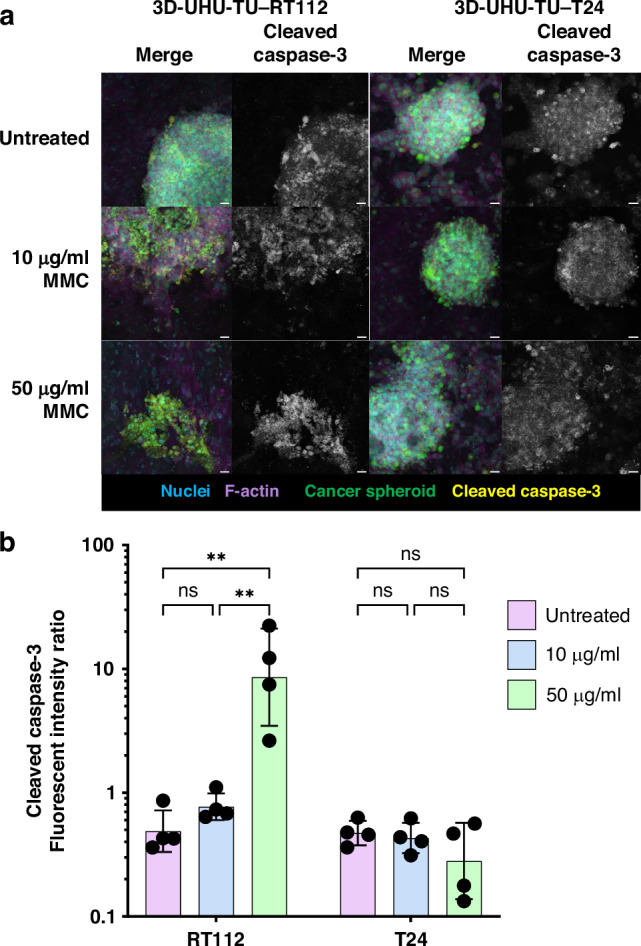
Automatic image analysis quantification of MMC treatment of 3D-UHU-TU models. **a** Representative examples of cleaved caspase-3-stained samples used for image analysis and quantification. Merged images are average intensity Z-projects and stained as follows: nuclei=cyan; F-actin=magenta; cancer cells=green; cleaved caspase-3=yellow. Cleaved caspase-3 channel is the sum of Z-projects in greyscale. Scale bars = 20 µm. **b** Quantification of cleaved caspase 3 in cancer region of full 3D-UHU-TU models. Results plotted as fluorescent intensity ratio between cleaved caspase 3 divided by cell nuclei of each cancer region. **P = < 0.01, ns no significance, *N* = 4.

The LDH assay could not be deployed to assess cell death in the complete models because it is not possible to distinguish the effects stemming from the individual compartments. Therefore, to quantify cell death in the complete models and assess whether the cell damage could be ascribed to apoptosis, we used cleaved caspase 3 immunostaining and image analysis (Fig. 4a and Fig. S5). These experiments indicated that, at this time point, caspase-3 was activated in the RT112 model, and there was a significant increase in apoptotic cell death with 50 µg/ml MMC treatment compared with untreated and 10 µg/ml MMC treatment (Fig. 4b). However, the T24 model showed no significant increased expression of cleaved caspase-3 after MMC treatment.

#### Oncolytic virus co-localises and disaggregates cancer spheroids within 3D-UHU-TU models

As a second trial of the utility of 3D-UHU-TU for therapy testing, we turned to a less conventional treatment: Oncolytic Herpes Simplex Viruses (oHSV), which were designed to replicate solely in tumour cells [33]. oHSV relies on protein kinase R (PKR), which is active in healthy cells and inhibited in cancer cells. Deletion of the ICP34.5 gene in oHSV allows PKR-mediated blocking of viral replication in healthy cells, enabling cancer cell-specific replication [27]. GFP tagged oHSV viruses (oHSV-GFP) were incubated with the 3D-UHU-TU models at an MOI of 0.05 for 30 min in 100% urine, changed every day to simulate urination. Co-localisation and viral propagation were assessed using confocal microscopy.

**Fig. 5:**
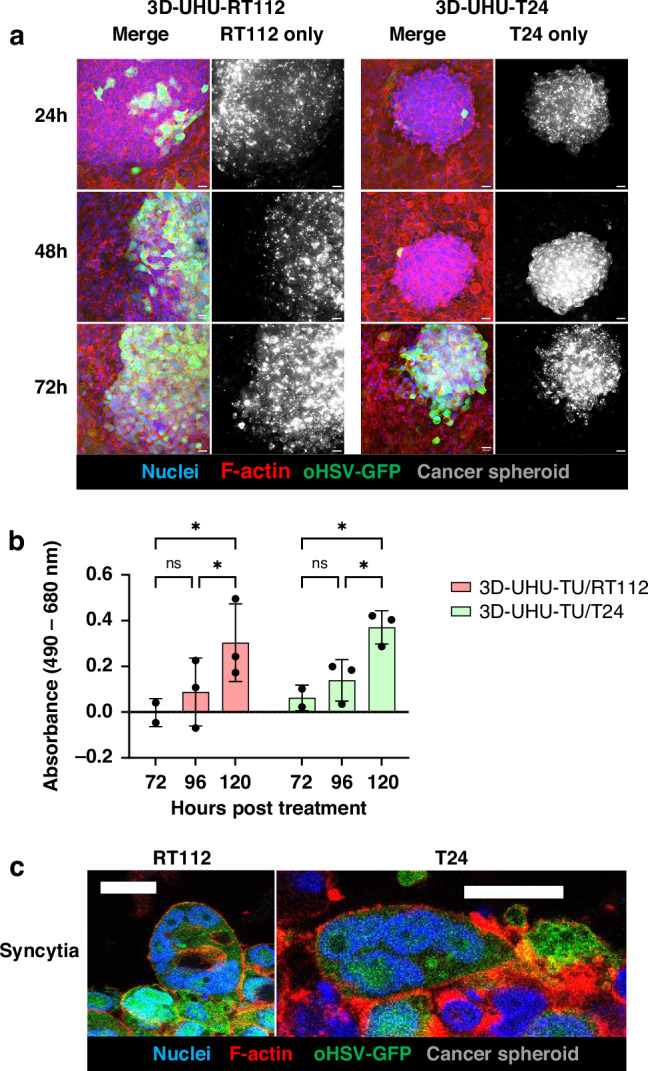
oHSV-GFP treatment of 3D-UHU-TU models. **a** Specific co-localisation of oHSV-GFP with RT112 and T24 cancer spheroids with minimal to no viral infection in healthy 3D-UHU component. oHSV-GFP propagated in cancer spheroids over 72 h post treatment (PT). Merged images are shown as summed z-projects. Scale bars = 20 µm. **b** Percentage cytotoxicity of 3D-UHU-TU models over 120 h PT. *P = < 0.05. Data are presented as mean averages of 3 independent experiments for 96 and 120 h and 2 independent experiments for 72 h. **c** Syncytia were also observed after 72 h PT in both cancer spheroids typical of fusogenic viruses. Scale bars = 20 µm. Images are representative of 3 biological replicates unless stated. For all merged images, nuclei=blue; F-actin=red; oHSV-GFP=Green. Cancer spheroid=greyscale (shown separately).

oHSV-GFP co-localised to RT112 and T24 cancer spheroids and propagated over 72 h (Fig. 5a), with minimal to no infection in the healthy 3D-UHU component. Virus propagation was more rapid in RT112 cancer spheroids than in T24, but essentially all cancer cells of both models were infected after 72 h (Fig. 5a). To test for cytotoxicity, oHSV-GFP was tested initially in cancer spheroids alone. Cell death increased over 96 h in both RT112 spheroids but not in T24 spheroids (Fig. S6A). Disaggregation of both cancer spheroid types was observed after 96 and 120 h in both spheroid types (Fig. S6B).

When tested on the complete 3D-UHU-TU models, cancer-specific cytotoxicity was observed after 96 h with significant increase in cytotoxicity observed in both RT112 and T24 3D-UHU-TU models after 120 h (Fig. 5b). To confirm that the cytotoxicity stemmed from the cancer component specifically, normal 3D-UHU models were treated with oHSV-GFP. No oHSV-GFP was observed in 3D-UHU after 144 h (Fig. S7). Syncytia, common in fusogenic viruses, was observed after 72 h. in both cancer spheroid types (Fig. 5c), suggesting that the observed cell death in the cancer component was caused by oHSV-GFP.

## Discussion

We developed a 3D hybrid model (3D-UHU-TU) that supports bladder cancer spheroids in a physiologically relevant urinary environment consisting of a healthy human urothelium bathed apically in 100% urine for up to 6 days. This platform preserves key phenotypic traits of low-grade (RT112) and high-grade (T24) disease, including differential migratory behaviours and biomarker expression, supporting its biological relevance. The 3D-UHU model has previously been utilised for research into urinary tract infection [21, 22]. 3D-UHU-TU represents the first significant advancement in adapting this model to simulate another urological disease: bladder cancer. Not only is the 3D-UHU-TU model established in a healthy urothelial and urine-dependent environment, allowing for a more physiologically accurate tumour microenvironment, but it can also incorporate tumour spheroids from different origins–in this case, both low- and high-grade 3D bladder cancer spheroids were represented as a proof-of-principle. As there are differences in mouse and human urine [40], we used 100% human urine to ensure the model was more physiologically relevant and would translate more faithfully to the human tumour microenvironment and its treatment responses.

Characterisation of the 3D-UHU-TU models revealed them to largely express the expected markers in the correct spatial environment. It is worth mentioning that growth and implantation of larger spheroids (≥400 µm) is also feasible (Fig. S1), so there is scope to assess spheroids that incorporate a hypoxic and/or necrotic core [41]. However, due to imaging limitations, our current study focused on multiple micro-spheroids derived from RT112 and T24 cell lines.

Clinical diagnoses of bladder cancer using immunohistochemistry staining usually involve GATA3 [42], CK7 and CK20 [43]. The loss of GATA3 expression has been linked to higher-grade disease [44]. A tissue microarray study of 2710 bladder tumours found GATA3 to be significantly more prevalent in non-invasive Ta stage tumours (98% of 790 tumours), with a marked reduction in T2 to T4 stage tumours (59.8% of 1223 tumours) [30]. Since RT112 is a low-grade T2-stage cancer cell line, we anticipated GATA3 expression, and indeed, the 3D-UHU-TU/RT112 model demonstrated GATA3 expression in the spheroid component. In contrast, the 3D-UHU-TU/T24 model, consistent with the characteristics of a high-grade cancer cell line, did not exhibit GATA3 expression in the spheroid component.

CK7 and CK20 expression were analysed in another microtissue array study in various grades and stages of urothelial carcinoma across 1755 samples [45]. For CK7, in Ta stage and low-grade urothelial carcinoma, CK7 was expressed in 98.7% of 177 tumours. In 3D-UHU-TU/RT112, which models these types of tumours, CK7 was consistently expressed throughout. In high-grade urothelial carcinoma, CK7 was shown to be expressed in 86.3% of 1207 tumours [45], and in our 3D-UHU-TU/T24 model, we observed expression of CK7.

CK20 is useful in determining urothelial differentiation in a clinical context; however, it is highly dependent on case selection. Expression of CK20 is reported to be lower in low-grade cancers (52–66%) compared with high grade cancers (83–100%) [31]. Expression in the normal urothelium is primarily associated with the umbrella cell layer as a marker for cell differentiation and maturation [31]. Although CK20 has previously been shown to be expressed in the superficial umbrella cells of 3D-UHU by immunofluorescence analysis [21], we did not observe CK20 expression via IHC in the normal margins of any of our models. Positive staining controls were employed for CK20 expression, confirming no technical issues. Assessing expression with immunofluorescence microscopy on the transverse plane may be more optimal for CK20. Alternatively, the absence of CK20 in the urothelium component may be due to co-culture effects altering expression in adjacent cells, shedding of the top layer during the co-culture, or the IHC sample preparation process.

Cadherin switching in our models align with clinical profiles of NMIBC and MIBC, suggesting that the 3D-UHU-TU can recapitulate key features used in pathological grading. In our experiments, RT112 spheroids incorporated into 3D-UHU did exhibit some migratory cells, but strongly expressed E-Cadherin, consistent with their classification as a low-grade cancer model. Conversely, T24 spheroids incorporated into 3D-UHU displayed a more frequent migratory phenotype and had an increased expression of N-Cadherin, consistent with their high-grade cancer classification.

Expression of MMPs is also a relevant aspect to consider. Kudelski et al. examined their expression in low- and high-grade cancer tissue alongside healthy marginal tissue taken from the same patient. They found that MMPs were present in all tissue types regardless of grade and disease status, but MMP-9 expression was enhanced compared with MMP-2, particularly in higher-grade tissues [46]. Our models demonstrated expression of both MMP types in the tumour component, alongside several migration types, with RT112 spheroids displaying amoeboid and mesenchymal phenotypes and T24 spheroids displaying mesenchymal phenotype only. These are consistent with the variety known to occur [36, 47] and demonstrate the dynamic and versatile nature of 3D-UHU-TU. It should also be noted that we did not observe an invasion phenotype in our higher-grade model. Invasion is defined as the migration of cells through a tissue [48], which was not observed with the higher grade model, where the cancer cells migrated on the surface of the urothelium. However, it is possible that more time is required for invasion to occur, or that our model is lacking some component that might facilitate this. Enhancing the model to incorporate deeper layers of the bladder (e.g. lamina propria and/or muscle microtissue) alongside more time in co-culture (as seen in ref. [49]) might allow us to model invasion in future 3D-UHU-TU models.

As the 3D-UHU-TU model is maintained in a 100% urine environment, it was important to test the effect of the urine on cancer spheroid proliferation. The Ki-67 data from the low-grade RT112 spheroids within 3D-UHU-TU matched their proliferative nature seen in growth curves measured from individual spheroids growing in microcavity plates in their preferred culture medium. In contrast, Ki-67 expression of the high-grade T24 spheroids was low within the main body of the spheroid, yet high in migratory T24 cells that had actively detached from the spheroid (Fig. S3). Additionally, cell nuclei appeared morphologically normal. Taken together with Ki-67 expression, these data support viability at this timepoint in both spheroid types. Given that Ki-67 has been implicated in bladder cancer metastasis [50], this result is consistent with that role. In the healthy 3D-UHU component, proliferation was also seen in the basal layers and some intermediate cells. This was expected, as the transitional urothelium regenerates from the basal membrane upwards, with progenitor basal cells responsible for replacing cells that have matured and/or exfoliated. In addition, rapidly proliferating intermediate cells play a crucial role in establishing the multiple layers of the urothelium [32].

To assess 3D-UHU-TU as a testbed for therapies, we first used the common post-operative chemotherapeutic, MMC. DNA fragmentation was seen in all treated RT112 spheroids, which suggests that MMC did induce apoptosis. In the absence of phagocytic cells responsible for clearing apoptotic bodies–as is the case in the 3D-UHU-TU model – secondary necrosis is likely to occur [51], which was morphologically evident in both RT112 and T24-based models and explains the LDH release we observed in the separate components. In the full 3D-UHU-TU models, we quantified apoptotic cell death using a novel imaging analysis ImageJ macro called ‘FluorMask3D’. In the RT112 component, there was a significant increase in cleaved caspase expression, particularly in the highest tested dose (50 µg/ml), whereas in the T24 component, there was no difference after MMC treatment. It is possible earlier timepoints could pinpoint apoptosis under the same treatment conditions with secondary necrosis present [51]. We also observed that the 3D-UHU-TU model can highlight differences in MMC treatment between low- and high-grade cancer. MMC is usually used for treatment of NMIBC which is typically low-grade [52], and not for high grade cancers where more radical treatments would be implemented. This ability of the 3D-UHU-TU model to highlight differences in therapy efficacy could be useful for screening individual patient responses to prospective therapies.

We also investigated a targeted therapy of oncolytic herpes simplex virus (oHSV) designed to replicate only in tumour cells [27]. There has been increasing interest in intravesical viral therapy for superficial NMIBC. Recently, two non-coding viral vectors and one oncolytic adenovirus have been approved by the FDA [53]. oHSV-GFP is a fusogenic virus which can enhance virus transmission between cells. This is advantageous as one problem with oncolytic viruses is limited transmission through the tumour microenvironment [54]. In our model, syncytia were present (Fig. 5c) after infection with oHSV-GFP, alongside cancer spheroid damage and cytotoxicity (Fig. 5b). To model physiological conditions, urine was changed at each time point to mimic urination. Even with the resulting decrease in viral load, we still observed cancer cell disaggregation and cell death, showing that oHSV-GFP can persist despite simulated emptying of the bladder. Together, these experiments demonstrate that a more targeted cancer therapy could be tested on the 3D-UHU-TU model, and that the simultaneous presence of healthy and cancer components were useful for highlighting the therapy’s tumour-specificity.

Several other interesting systems have been reported in the literature that model bladder cancer in a more human micro-environment. For example, Booth et al. de-cellularised urothelial stroma from surgeries, then re-populated this foundation with stratified cancer cells [55]. This study successfully combined these two components and importantly, recreated the 3D architecture needed for a more complex bladder cancer model. However, as the model lacked the normal epithelial cells of the urothelium, which likely also affect the tumour micro-environment, it is rather different from our model. Ringuette Goulet et al. successfully established a bladder cancer model which incorporated healthy urothelial and cancer components. This included stroma and urothelial cells from normal bladder biopsies that were grown and differentiated in culture. They then grafted different grades of cancer spheroids onto the healthy component to create the ‘tissue engineered 3D BCa model’ [49]. This model adds the complexities of the stromal compartment (further enhanced with induced cancer-associated fibroblasts [56]) with a fully stratified urothelium, which our model does not. It also shows its efficacy for MMC treatment responses and physiologically relevant cell behaviour. However, it lacked the full characterisation that our 3D-UHU model has undergone [21]. Specifically, we confirmed that all three expected cell types were present, alongside uroplakins, which are essential to confirm mature umbrella cell fate, as well as the GAG layer and other key markers such as cytokeratins and E-cadherin. Crucially, the presence of 100% human urine in our urine-tolerant model is unique and makes it more physiologically relevant to treatment testing. It is worth mentioning that our 3D-UHU model uses commercially available components, and is relatively easy to manipulate and use. In our view, all models offer advantages and disadvantages, and it is beneficial for the community to use them in a complementary fashion while being aware of their relative strengths and weaknesses.

In conclusion, the 3D-UHU-TU model represents a new generation of in vitro urothelial bladder cancer model including a cancer component embedded in a healthy micro-environment, which is tractable and shows promise as a testing platform for therapies. This model could be used to determine treatment dose and schedule, and potentially test combinations with other treatments. Like all models, 3D-UHU-TU does have some inherent limitations, for example the lack of a blood supply, deeper tissue layers or an immune system. On the other hand, in recent years, great strides have been made in incorporating more complexity into micro-physiological systems (reviewed in [57]), suggesting that the 3D-UHU-TU prototype could be used a foundation for developing more advanced iterations. One exciting possibility, which we are actively pursuing, is the incorporation of patient-derived tumour spheroids in place of cell lines, potentially allowing a personalised medicine approach to drug screening. Also, as in vitro cell-based model technologies become more evolved, 3D-UHU-TU with such patient material could serve as a tool to help further understand the disease at the molecular level.

## Supplementary information

Supplementary materials

## Data Availability

All data needed to evaluate the conclusions in the paper are present in the paper and/or the Supplementary Materials. The ImageJ macro “FluorMask3D” is available from 10.5281/zenodo.19582036.
