# Supplementary material for: An advanced in vitro bladder cancer model integrating bladder cancer spheroids into a healthy human urothelium for preclinical therapeutic testing
Source: Br J Cancer. 2026 Jun 2;135(5):697–707. doi: 10.1038/s41416-026-03476-0 (PMC13478310; doi:10.1038/s41416-026-03476-0)
Supplement: Supplementary file 1 [file 41416_2026_3476_MOESM1_ESM.docx]

**Supplementary materials**

**Table S1: Antibody details**

| **Target/Stain** | **Host** | **Company** | **Product code** | **Concentration/ Dilution used** | **Other details** |
| --- | --- | --- | --- | --- | --- |
| **Primary Antibodies** | | | | | |
| **E-Cadherin** | Mouse | Invitrogen | 13-1700 | 10µg/ml | Monoclonal |
| **N-Cadherin** | Rabbit | Invitrogen | PA5-29570 | 1µg/ml | Polyclonal |
| **Ki-67 (IF)** | Mouse | Invitrogen | 50-5699-82 | 1µg/ml | Monoclonal;  Anti-human eFluor™ 660 conjugate |
| **Ki-67 (IHC)** | Mouse | Leica | PA0230 | N/A | Bond RTU; Monoclonal |
| **MMP-2** | Mouse | Invitrogen | 436000 | 2µg/ml | Monoclonal |
| **MMP-9** | Mouse | Invitrogen | MA5-15886 | 1:200 | Monoclonal |
| **CK7** | Mouse | Leica | PA0942 | N/A | Bond RTU; Monoclonal |
| **CK20** | Mouse | Leica | PA0022 | N/A | Bond RTU; Monoclonal |
| **GATA3** | Mouse | Sigma | 390M-14 | 1:300 | Monoclonal |
| **Phalloidin-555** | - | Invitrogen | A30106 | 0.04µM/ml (1:500) | - |
| **Phalloidin-647** | - | Invitrogen | A30107 | 0.04µM/ml (1:500) | - |
| **DAPI** | - | Invitrogen | D1306 | 1µg/ml | - |
| **Secondary Antibodies** | | | | | |
| **Anti-Mouse 488** | Goat | Invitrogen | A11001 | 10µg/ml | - |
| **Anti-Rabbit 488** | Goat | Invitrogen | A11034 | 10µg/ml | - |
| **Anti-Mouse 647** | Goat | Abcam | ab150115 | 10µg/ml | - |
| **Anti-Rabbit 647** | Goat | Abcam | Ab150079 | 10µg/ml | - |

RTU = Ready to use; IF = Immunofluorescence; IHC = Immunohistochemistry

Figure S1: Light Sheet micrographs of RT112-GFP spheroids further boosted with GFP antibody staining (green) incorporated onto 3D-UHU (A) after 24 hours in 20% FBS; (B) after 24 hours in 20% FBS with intercalation of cancer cells (white arrows); (C) after 24 hours in 10% FBS with intercalation of cancer cells (white arrows). Blue strand is an unknown artefact. Membrane is stained with WGA-555 (red). Scale bar=100 µm

Figure S2: (A) 3D-UHU model expressed E-Cadherin in differentiated umbrella cells. Image is Z-project at max intensity. Scale bar = 50µm. 3D-UHU has previously been shown to express E-Cadherin (21). MMP-2 (B) and MMP-9 (C) expression of RT112 and T24 spheroids integrated onto the 3D-UHU model 24 hours post-integration (3D-UHU layer is below the plane of focus). MMP-2 and -9 (Red, Panels B and C) were expressed in the correct areas with different migration phenotypes of cells migrating from the main spheroid. In RT112 spheroids, migration phenotypes included amoeboid (white arrows) and mesenchymal (yellow arrows). There is also evidence of collective migration (36) (magenta arrows). In T24 spheroids, there were more migrating cells (green arrows) as expected of a higher-grade cancer. There was also a mesenchymal migration phenotype present (yellow arrow). Scale bars = 50µm.

Figure S3: Expression of Ki-67 proliferation marker in 3D-UHU-TU/T24. (A) Split channels of immunofluorescence with separate T24 spheroid and 3D-UHU after 2 days in co-culture. Cancer spheroid (CS) is located to the right of the orange dotted line. Expression in migrating cells was observed but limited expression in the main spheroid. Scale bars = 50µm.

**Nuclei	F-Actin	Cancer**

Figure S4: 3D-UHU-TU models treated with saline for two hours. No damage is observed and cancer spheroids remain intact. Models were recovered with fresh urine for 24 hours post treatment, fixed, stained and imaged as mentioned before. Nuclei = blue, F-actin = magenta and cancer cells = green. Scale bars = 20µm.

Figure S5 – Image analysis “FluorMask3D” workflow description. (i) First the background was measured from slide 0 and subtracted from the green channel corresponding to the cancer spheroid. Then, the corrected image was filtered with a Gaussian Blur and a reduction of pixels to 728x728 was performed; (ii) Next, a binary mask was made for the whole stack (e.g. 70 slices) of the image (white = 255, black = 0); (iii) This mask was then overlayed on the fluorescence image (background corrected) to calculate the total area of the mask and its intensity signal for each slice; (iv) To find the region corresponding only to the cancer spheroid, the total area from each slice was plotted against the slices. This graph generally follows an asymmetric double sigmoid function. The steeper points on each side represent the beginning and end of the cancer spheroid; these were found by the FIJI macro and these slides (e.g. 6-61) are designated as the cancer region. (iv) The steps (i)-(iii) were repeated with the cleaved caspase-3 yellow channel and with the cyan nuclei. Once the mask was created, it was cropped using the cancer region slides (e.g. 6-61) then in this region the mean fluorescence for the masked area in each slice was measured and then summed together to produce the fluorescence value for each channel. The ratio of yellow/cyan was calculated as the relative fluorescence of the cleaved caspase-3 indicating death of cells within the cancer spheroid.

**Orthogonal Merge**

**RT112**

**Orthogonal Merge**

**T24**

**C**

Figure S6: Effect of oHSV-GFP on cancer spheroids. (A) Spheroid cytotoxicity of cancer spheroids alone from 24-96 hours post treatment. LDH cytotoxicity assay was performed in triplicate with numbers blank-subtracted and normalised to untreated control. Results are from 1 biological replicate with 3 technical replicates. ***p=<0.001; ****p<0.0001. (B) Morphological changes to spheroids treated with oHSV-GFP vs. untreated over 96 hours. Disaggregation observed from 48 hours in RT112 spheroids and 96 hours in T24 spheroids (purple arrow) compared with untreated spheroid solid edge (black arrow). (C) Disaggregation was observed in 3D-UHU-TU models (blue arrows) caused by oHSV-GFP with syncytia observed (white arrows) up to 120 hours. Untreated controls are from 144 hours post treatment. Images are representative of 3 independent biological replicates. Scale bars = 20µm.

Figure S7: oHSV-GFP treatment of healthy 3D-UHU from 72-144 hours. No oHSV-GFP was observed in treated 3D-UHU (top), nor with untreated controls (bottom). Nuclei=blue; F-actin=red; oHSV-GFP=green. Scale bars = 20µm.
